# Identifying extra pulmonary vein targets for persistent atrial fibrillation ablation: bridging advanced and conventional mapping techniques

**DOI:** 10.1093/europace/euaf048

**Published:** 2025-03-12

**Authors:** Alexander J Sharp, Michael T Pope, Andre Briosa e Gala, Richard Varini, Abhirup Banerjee, Timothy R Betts

**Affiliations:** Institute of Biomedical Engineering, Department of Engineering Science, University of Oxford, Oxford OX37 DQ, UK; Cardiology Department, John Radcliffe Hospital, Oxford University Hospitals NHS Foundation Trust, Oxford OX3 9DU, UK; Cardiology Department, Southampton General Hospital, University Hospital Southampton NHS Foundation Trust, Southampton, SO16 6YD, UK; Cardiology Department, Southampton General Hospital, University Hospital Southampton NHS Foundation Trust, Southampton, SO16 6YD, UK; Cardiology Department, John Radcliffe Hospital, Oxford University Hospitals NHS Foundation Trust, Oxford OX3 9DU, UK; Institute of Biomedical Engineering, Department of Engineering Science, University of Oxford, Oxford OX37 DQ, UK; Cardiology Department, John Radcliffe Hospital, Oxford University Hospitals NHS Foundation Trust, Oxford OX3 9DU, UK

**Keywords:** Persistent atrial fibrillation, Catheter ablation, Charge density mapping, Electroanatomic voltage mapping, Low-voltage areas, Conduction velocity

## Abstract

**Aims:**

Advanced technologies such as charge density mapping (CDM) show promise in guiding adjuvant ablation in patients with persistent atrial fibrillation (AF); however, their limited availability restricts widespread adoption. We sought to determine whether regions of the left atrium containing CDM-identified pivoting and rotational propagation patterns during AF could also be reliably identified using more conventional contact mapping techniques.

**Methods and results:**

Twenty-two patients undergoing *de novo* ablation of persistent AF underwent both CDM and electroanatomic voltage mapping during AF and sinus rhythm with multiple pacing protocols. Through the use of a left atrium statistical shape model, the location of distinctive propagation patterns identified by CDM was compared with low-voltage areas (LVAs) and regions of slow conduction velocity (CV). Neither LVA nor CV mapping during paced rhythms reliably identified regions containing CDM propagation patterns. Conduction velocity mapping during AF did correlate with these regions (ρ = −0.63, *P* < 0.0001 for pivoting patterns; ρ = −0.54, *P* < 0.0001 for rotational patterns). These propagation patterns consistently occurred in two specific anatomical regions across patients: the anteroseptal and inferoposterior walls of the left atrium.

**Conclusion:**

Mapping techniques during paced rhythms do not reliably correspond with regions of CDM-identified propagation patterns in persistent AF. However, these propagation patterns are consistently observed in two specific anatomical regions, suggesting a predisposition to abnormal electrophysiological properties. While further research is needed, these regions may serve as promising targets for empirical ablation, potentially reducing the reliance on complex mapping techniques.

## Graphical Abstract

**Graphical Abstract:**
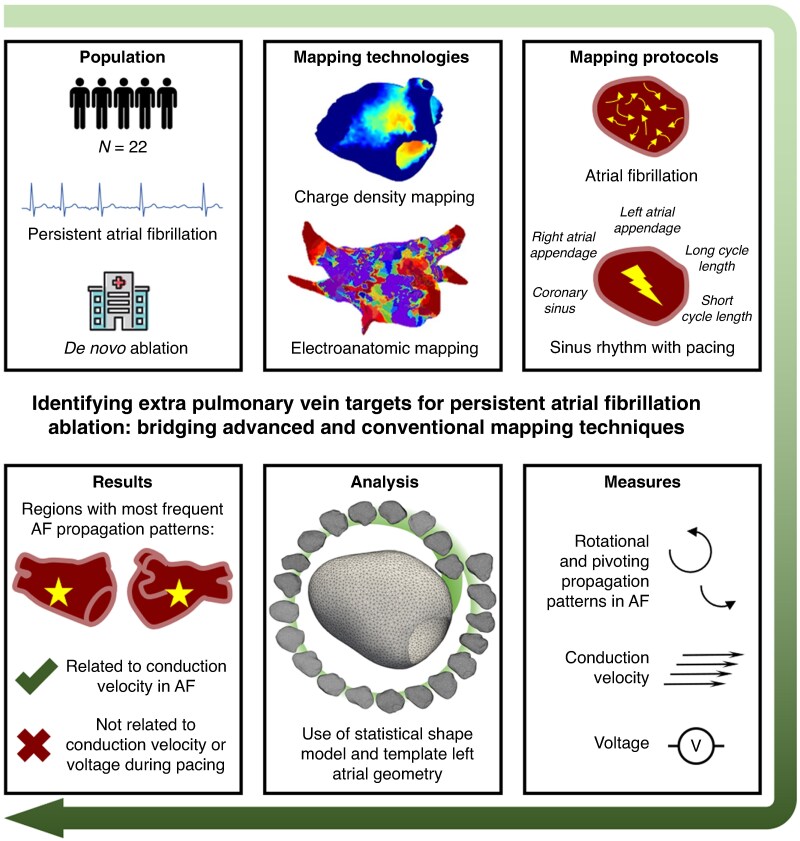

**What’s new?**

- In patients with persistent atrial fibrillation (AF), we identify two consistent anatomical regions of the left atrium (anteroseptal and inferoposterior walls) where pivoting and rotational propagation patterns occur during AF.
- Neither low-voltage areas, nor conduction velocity mapping during sinus rhythm and pacing, reliably identified these regions.
- Conduction velocity mapping during AF can identify these regions but has limited feasibility with current contact mapping technologies.
- Our findings bridge the gap between advanced mapping techniques and practical, widely applicable ablation strategies for persistent AF.

## Introduction

Since the landmark study by Haissaguerre *et al*.^1^ identified the pulmonary veins (PV) as critical triggers, pulmonary vein isolation has formed the cornerstone of catheter ablation treatment for AF.^2,3^ Being a purely anatomical approach has led to the widespread availability of this treatment using a variety of modalities. While highly effective in reducing AF burden and improving quality of life, outcomes in persistent AF remain suboptimal, with recurrence rates in excess of 40%.^4–6^

A significant number of patients with persistent AF undergo additional ablation beyond PVI in an attempt to achieve long-term freedom from arrhythmia. This has driven the development of adjunctive ablation strategies, broadly categorized as empiric or individualized approaches. No strategy employing empirical isolation of atrial structures has proven superior to PVI alone in randomized controlled trials, despite hints at potential utility in specific subgroups.^7–9^ Individualized strategies have shown promise but with limited widespread adoption. Low-voltage area (LVA) ablation, targeting presumed fibrotic regions, has demonstrated modest benefits in some patients, although not all individuals exhibit significant LVAs.^10,11^ Similarly, conduction velocity (CV) mapping to identify regions of slow conduction has shown potential, but standardized methodologies and clear ablation targets remain elusive.^12–14^

Recent advances in mapping technologies, particularly non-contact charge density mapping (CDM), have enabled high-resolution, whole-chamber assessment of AF dynamics. Both the UNCOVER AF^15^ and RECOVER AF^16^ trials have demonstrated promising results, using CDM to identify and target recurring propagation patterns. These propagation patterns, which include pivoting and rotational wavefront behaviours, are believed to originate from pathophysiological substrates, for example regions of fibrosis and heterogeneous conduction.^16^ However, the specialized nature of CDM limits its widespread applicability in clinical practice.

Given these challenges, there remains a critical need for easily applicable individualized or empirical approaches that offer clinically significant improvements in ablation outcomes for persistent AF. Ideally, such approaches would leverage more widely available mapping technologies while still capturing the essential elements of arrhythmogenic substrate identified by advanced mapping techniques.

In this study, we sought to determine whether regions containing pivoting and rotational propagation patterns identified by CDM during AF could be reliably detected using more conventional approaches, comprehensively assessing the use of voltage and CV across a range of mapping protocols. Additionally, we evaluated whether these patterns localized to consistent anatomical regions across patients, potentially supporting an empirical, anatomically guided ablation strategy that could bridge the gap between individualized mapping and practical, widely applicable treatment strategies.

## Methods

### Study population

The present study is a retrospective analysis using a cohort derived from CASDAF-HD (clinicaltrials.gov NCT04229472). This single-centre, non-randomized, mechanistic study recruited 31 patients scheduled for an elective first-time catheter ablation of AF between July 2022 and November 2023. Twenty-two patients within this study underwent mapping of persistent AF with both CDM and electroanatomic voltage mapping (EAVM). All patients consented to their data being used in further studies. The investigation conformed to the principles outlined in the Declaration of Helsinki, and the original study protocol was approved by London-Surrey Research Ethics Committee (REC reference 20/LO/0150).

### Electrophysiological study procedures

Procedures were performed under general anaesthetic. Antiarrhythmic medications (except amiodarone) were stopped a minimum of 5 days before the procedure. Venous access was obtained via bilateral femoral vein puncture under ultrasound guidance. Heparin boluses were administered prior to trans-septal puncture, followed by a continuous heparin infusion to maintain ACT >350 ms.

Voltage and CV measurements can vary depending on rhythm, cycle length (CL), and wavefront direction.^12–14,17–19^ To ensure a comprehensive assessment that would identify the potential utility of different mapping protocols, we considered all these variables. Mapping was performed prior to any ablation, initially in the patient’s presenting rhythm before AF termination with direct current cardioversion, or induction with atrial burst pacing. Sinus rhythm mapping was performed during bipolar pacing with a repeating four-beat drive train at 800 ms CL, followed by a single extrastimulus at 300 ms (or shortest captured) CL, using the minimum pacing threshold necessary for reliable capture. Pacing was performed from up to three different locations: mid-coronary sinus (CS), left atrial appendage (LAA), and right atrial appendage (RAA).

CDM using the AcQMap system (Acutus Medical, CA) has been previously described.^15,16,20–22^ Once positioned within the left atrium, catheter-based ultrasound was used to reconstruct the endocardial surface, and non-contact unipolar recordings of electrical activity were collected. During AF, a recording of global chamber electrical activity was produced with the catheter stationary in the middle of the chamber. During sinus rhythm (SR), a multi-position approach increased mapping resolution by bringing the catheter into close proximity with all endocardial surfaces.^23^

High-density EAVM was performed using the Advisor HD Grid and Ensite Precision Cardiac Mapping System (Abbott Laboratories, IL, USA); by using omnipolar mapping technology, we aimed to mitigate the impact of wavefront direction on measured voltage.^12,24^ Interpolation threshold was set to 7 mm. For mapping performed during SR, initial maps were produced for 800 ms CL pacing, with TurboMap used to retrospectively generate maps from extrastimulus pacing.

### Data analysis

#### Charge density mapping derived propagation patterns and conduction velocity

Analysis of CDM was performed offline using AcQMap 8.5 software (Acutus Medical, CA). Limited post-processing of left atrial geometries involved the removal of the PV and the LAA. This was followed by closing the surface meshes and excluding the mitral valve (MV), resulting in geometries that featured a single opening and contained approximately 3000 vertices. The removal of non-conducting structures is established practice,^16^ and this post-processing step enabled the effective alignment of left atrial geometries from different individuals during subsequent analyses.

Pivoting and rotational propagation patterns during AF were defined using AcQTrack software, which detected propagation exceeding 90 and 270 degrees of rotation around a single point, respectively. These patterns were quantified as occurrences per second at each vertex.^15,16,22^

To calculate local activation times (LAT) from CDM signals, a neighbouring region of approximately 5 mm was defined around each vertex of the left atrial anatomy. A singular value decomposition allowed the first two principal components to be calculated, and this region to be projected onto a 2D plane defined by these components. Gradient of activation was estimated using a third-order polynomial surface fit, which is inverted to estimate CV at each vertex.^25^ This approach has been previously validated against electroanatomic mapping-derived LAT.^26^

For recordings during AF, a 20 s segment was analysed; this duration was selected to balance the need for a reliable representation of AF propagation patterns with efficient data analysis and is supported by previous work.^27^ Following manual identification and exclusion of ventricular ectopics, a QRS subtraction algorithm was applied. The resultant signal was used by the CDM algorithm to derive whole-chamber propagation. As propagating wavefronts may pass each vertex multiple times during the 20 s segment, multiple values for CV were calculated at each vertex.

For multi-position SR recordings, signals were ‘binned’ automatically according to CL as measured from a decapolar catheter within the CS. This allowed signals from long and short CL pacing to be separated, and for signals from each position in the chamber to be combined to determine global chamber electrical activity. Subsequently, for each pacing protocol, a single CV value was calculated at each vertex.

#### Template left atrial geometry

To facilitate comparison of spatial distributions between individuals and mapping modalities, a template left atrial geometry was constructed from all 22 CDM geometries. Using ShapeWorks 6.4,^28^ geometries were first normalized to a common scale and then aligned using an iterative closest point (ICP) algorithm. Subsequently, a particle-based shape modelling approach was employed to calculate correspondence points across the geometries. This method enabled the accurate computation of the template shape.

Within MATLAB R2022b (The MathWorks, Natick, MA, USA), individual patients’ CDM left atrial geometries were aligned with this template geometry using an ICP algorithm followed by centroid alignment. Technical differences in geometry creation meant the alignment of EAVM geometries required additional steps; geometries were normalized for scale prior to ICP registration, and the ICP algorithm was enhanced with additional weighting to specifically ensure accurate alignment of the MVs. The accuracy of all registrations was confirmed through visual inspection and calculation of the mean distance between our template geometry and patients’ CDM and EAVM geometries. Subsequently, a nearest neighbour matching algorithm was used to apply values for CDM propagation pattern frequency, CV, and voltage, from individual geometries to the template geometry.

#### Statistical analysis

Continuous data are described as mean ± SD if normally distributed or as median (first—third quartile). Categorical data are described with absolute and relative frequencies. The Wilcoxon signed-rank test and Mann–Whitney *U* test were used when comparing non-normally distributed data from paired and independent groups, respectively. Data from all patients were used in each analysis where available. Population-wide spatial distributions used median values at each vertex of our template atrial geometry. Correlation analysis of CDM propagation pattern frequencies vs. CV depending on mapping protocol, used median binned plots for individual patients and Spearman’s rank correlation coefficient across the entire population. Statistical analysis was performed using R (version 4.3.1) and MATLAB. All statistical tests were two-sided, and *P* < 0.05 was considered statistically significant.

## Results

### Patient characteristics

The clinical and procedural characteristics of all patients are outlined in *Table 1*, with further clinical details presented in Supplementary material online, *Table S1*. The mean age was 63.8 ± 10.6 years and 64% were male. All patients had CDM performed during AF. 86, 55, and 55% had CDM performed during CS, LAA, and RAA pacing, respectively. 77% had EAVM during CS pacing, and 50% EAVM during AF. Differences in these percentages reflect data availability in the original study cohort, a key contributor to this being technical challenges related to rhythm stability. Achieving and sustaining SR was challenging in some individuals; CS pacing was performed first during data acquisition, which may account for the greater percentage of mapping completed during CS vs. LAA and RAA pacing.

**Table 1:** Clinical and procedural characteristics of the study population

Characteristics	Distribution
Age, years, mean ± SD	63.8 ± 10.6
Sex, *n* (%)	
Men	14 (64)
Women	8 (36)
BMI (kg/m^2^), mean ± SD	30.8 ± 5.2
CHA_2_DS_2_-VASc score, *n* (%)	
0	2 (9)
1	6 (27)
2	5 (22)
3	4 (18)
4	3 (14)
5	1 (5)
6	1 (5)
Prior antiarrhythmic drugs, *n* (%)	
Sotalol	17 (77)
Amiodarone	8 (36)
Time from first diagnosed AF, years, median (IQR)	2.4 (1.5–3.0)
Ablation type, *n* (%)	
*De novo*	18 (82)
Retreatment	4 (18)
Rhythm at baseline, *n* (%)	
AF	11 (50)
SR	11 (50)
Mapping performed, *n* (%)	
CDM during AF	22 (100)
CDM during CS pacing	19 (86)
CDM during LAA pacing	12 (55)
CDM during RAA pacing	12 (55)
EAVM during CS pacing	17 (77)
EAVM during AF	11 (50)

AF, atrial fibrillation; BMI, body mass index; CDM, charge density mapping; CS, coronary sinus; EAVM, electroanatomic voltage mapping; IQR, interquartile range; LAA, left atrial appendage; RAA, right atrial appendage; SD, standard deviation; SR, sinus rhythm.

### Registration accuracy with template geometry

Averages of the mean distance between our template geometry and patients’ CDM and EAVM geometries were 2.7 ± 1.7 mm and 3.9 ± 2.7 mm respectively. Individual values are presented in Supplementary material online, *Table S2*, alongside visualizations of individual case registrations in Supplementary material online, *Figure S1*.

### Distributions of charge density mapping propagation patterns

**Figure 1:**
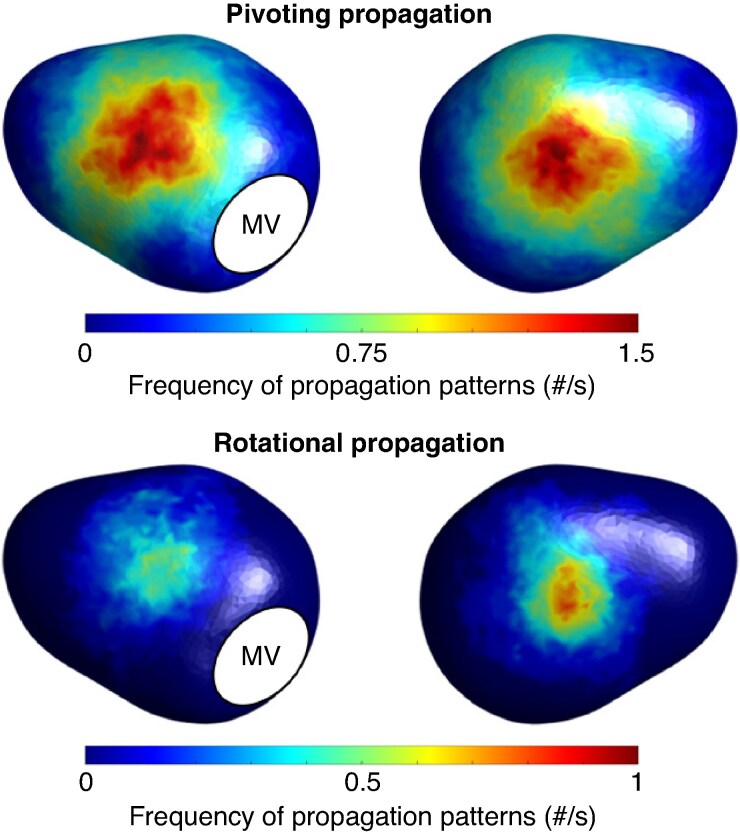
Anterior (left) and posterior (right) views of population-wide spatial distributions of CDM propagation patterns when applied to template left atrial geometry. MV, mitral valve.

*Figure 1* shows the population-wide spatial distributions of CDM propagation patterns during AF. Both pivoting and rotational propagation patterns were observed more frequently on anteroseptal and inferoposterior aspects of the left atrium. Results were similar in a sub-analysis excluding patients on amiodarone (see Supplementary material online, *Figure S2*).

### Distributions of low-voltage areas

**Figure 2:**
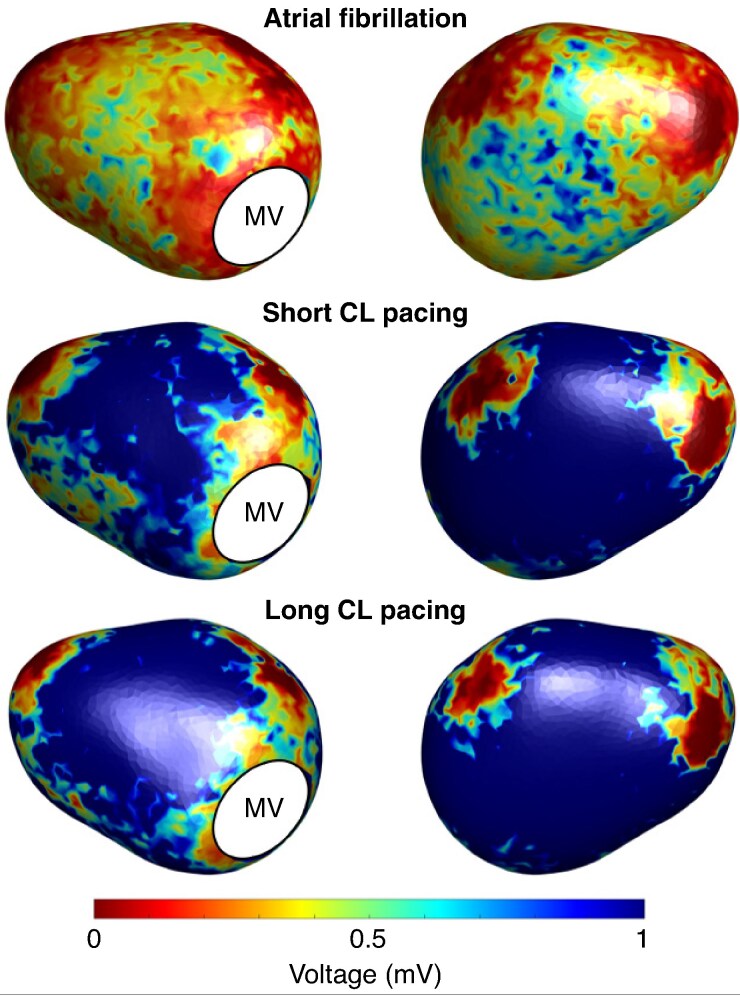
Anterior (left) and posterior (right) views of population-wide spatial distributions of voltage depending on mapping protocol when applied to our template left atrial geometry. CL, cycle length; MV, mitral valve.

*Figure 2* shows the population-wide spatial distributions of voltage identified during EAVM, depending on mapping rhythm. The lowest voltage regions were around the PVs, MV, and LAA, being an expected result of registration with our template atrial geometry. Outside of these regions, distribution of LVAs was visually similar in both paced maps, being primarily located in the septal region. There was extension of the LVAs observed in this region in short vs. long CL pacing, and this was supported by a lower global median voltage: 0.98 (0.50–1.31) mV vs. 1.07 (0.80–1.86) mV, *P* = 0.007. Global median voltage was lower still in AF [0.30 (0.23–0.49) mV, *P* = 0.0006 vs. short CL pacing] with a similar distribution pattern. Results were similar in a sub-analysis excluding patients on amiodarone (see Supplementary material online, *Figure S3*).

### Distributions of conduction velocity

**Figure 3:**
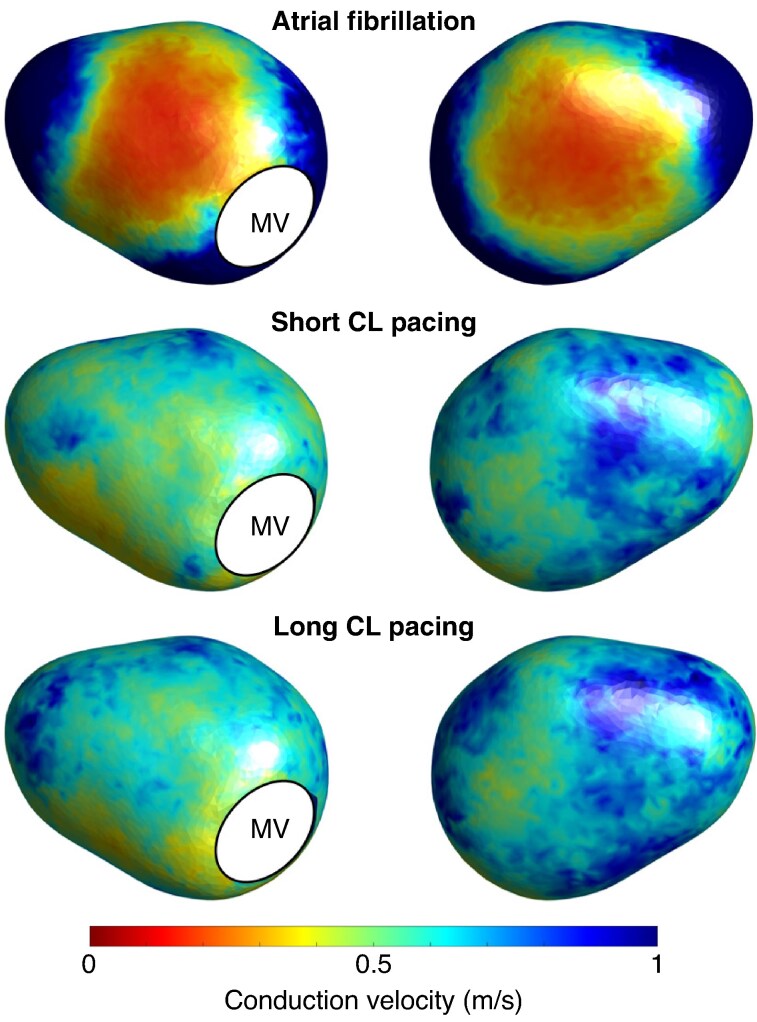
Anterior (left) and posterior (right) views of population-wide spatial distributions of conduction velocity depending on mapping protocol when applied to our template left atrial geometry. CL, cycle length; MV, mitral valve.

**Figure 4:**
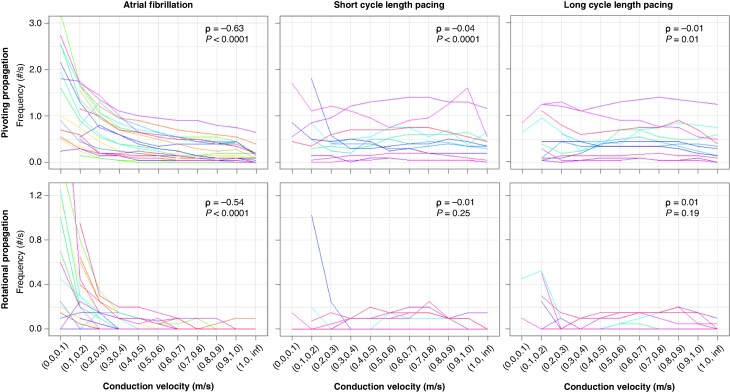
Relationships between conduction velocity and propagation patterns representing potential atrial fibrillation drivers. Coloured lines represent individual patients.

**Figure 5:**
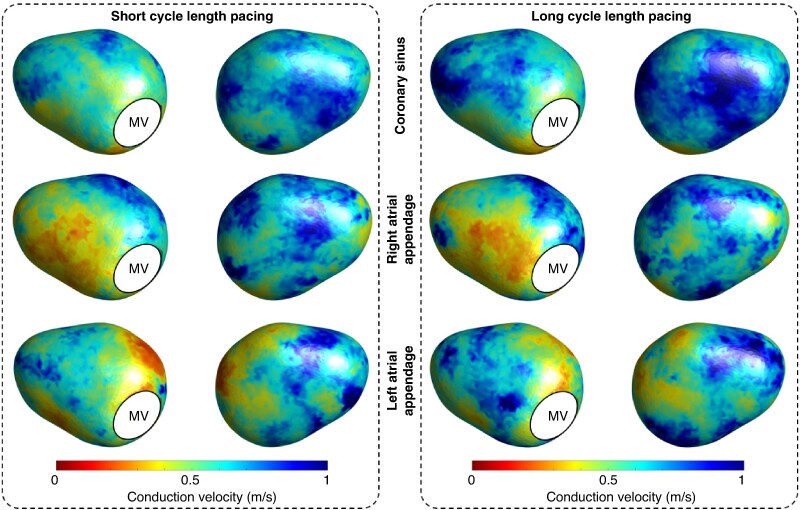
Population-wide spatial distributions of conduction velocity depending on pacing site when applied to our template left atrial geometry. MV, mitral valve.

To mitigate the impact of wavefront direction on CV during pacing, median values from all pacing sites were utilized in *Figures 3* and *4*, with *Figure 5* showing CV depending on each pacing site.

*Figure 3* shows the population wide spatial distributions of CV identified during CDM, depending on mapping rhythm. Regions of slow CV were seen on both the anteroseptal and inferoposterior aspects of the left atrium during AF in a visually similar distribution to CDM propagation patterns. Results were similar in a sub-analysis excluding patients on amiodarone (see Supplementary material online, *Figure S4*).

Quantitatively, *Figure 4* shows median binned plots of CV vs. frequency of CDM propagation patterns for individual patients, with Spearman's rank correlation coefficient calculated across the entire population. There was moderate correlation between slower CVs during AF and both pivoting and rotational propagation patterns (*ρ* = −0.63, *P* < 0.0001 and −0.54, *P* < 0.0001, respectively). The strength of all correlations between SR CV and CDM propagation patterns was very weak and essentially negligible (short CL pacing vs. pivoting propagation: ρ = −0.04, *P* < 0.0001; long CL pacing vs. pivoting propagation: *ρ* = −0.01, *P* = 0.01; short CL pacing vs. rotational propagation: ρ = −0.01, *P* = 0.25; long CL pacing vs. rotational propagation: *ρ* = 0.01, *P* = 0.19). *Figure 5* shows CV depending on each pacing site. During both short and long CL pacing, regions of slow conduction were most prevalent around the site of pacing, or Bachmann’s bundle in the case of RAA pacing.

### Presenting vs. induced atrial fibrillation

**Figure 6:**
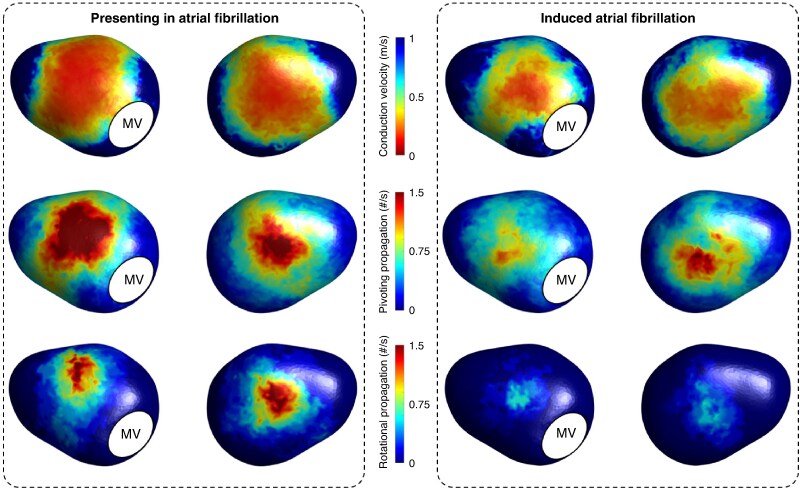
. Comparison of conduction velocity, and pivoting and rotational propagation pattern frequency during atrial fibrillation dependent on whether this was individuals’ presenting procedural rhythm or induced. MV, mitral valve.

*Figure 6* compares CV and CDM propagation patterns during AF, depending on whether this was a patient’s presenting procedural rhythm or induced. Distributions were similar across both cohorts, with primarily involvement of the anteroseptal and inferoposterior walls. Whilst not statistically significant, the magnitude of CV slowing and frequency of CDM propagation patterns were greater in those presenting in AF: global median value of CV 0.46 (0.39–0.68) m/s vs. 0.71 (0.44–0.84) m/s, *P* = 0.21; frequency of pivoting propagation patterns 0.60 (0.28–0.75) #/s vs. 0.40 (0.20–0.55) #/s, *P* = 0.47; frequency of rotational propagation patterns 0.10 (0–0.15) #/s vs. 0 (0–0.05) #/s, *P* = 0.06.

### Clinical outcomes

Three patients (14%) had confirmed AF recurrence during 1-year routine clinical follow-up that included clinic review, 12-lead ECG, and ambulatory monitoring where indicated:

- Case 4 presented with slow CV and pivoting propagation patterns during AF primarily localized to the anterior wall. Ablation included additional lesions on the anterior and posterior walls.
- Case 11 presented with slow CV and pivoting propagation patterns during AF across both the anterior and posteroinferior walls. Ablation included additional lesions on the anterior wall and roof.
- Case 20 presented with slow CV and pivoting propagation patterns during AF across both the anterior and posteroinferior walls and received PVI only.

Further details on individual AF recurrence outcomes and the locations of additional ablation lesions are provided in Supplementary material online, *Table S3*.

## Discussion

Our study provides important insights into the relationships between atrial substrate characteristics identified by various mapping modalities and propagation patterns observed during CDM of AF. The key findings of our investigation are: (i) neither LVAs nor CV mapping during paced rhythms reliably identified regions containing CDM propagation patterns; (ii) CV mapping during AF correlated with these regions but is not widely applicable with contact mapping systems; and (iii) CDM propagation patterns consistently occurred in two specific anatomical regions across patients.

### Charge density mapping propagation patterns

AF is driven by complex conduction dynamics, with high-density optical mapping and computational models supporting functional re-entry, in the form of rotors, as a key mechanism in its maintenance.^29,30^ When applied to clinical mapping, how rotors are defined and identified can vary significantly between modalities, leading to discrepancies in reported relevance. However, a recent meta-analysis of 10 randomized or case-controlled clinical trials^30^ found that rotor-guided ablation was associated with improved outcomes compared with conventional strategies, with an odds ratio of 0.53 (CI 0.40–0.69, *P* = 0.037).

In the context of CDM, rotational and pivoting wavefronts have been identified as significant propagation patterns that may reflect underlying re-entrant activity. The clinical relevance of CDM-derived propagation patterns has been demonstrated in several studies. Shi *et al*.^22^ compared outcomes in 45 patients undergoing PVI plus CDM-guided ablation with 80 propensity score-matched control patients who underwent PVI plus empirical posterior wall isolation; at 24 months, freedom from AF and atrial tachycardia was significantly higher in the CDM group (68% vs. 46%, *P* = 0.043). The UNCOVER AF^15^ multicentre trial, which included 129 *de novo* ablation patients, reported a 12-month, single-procedure freedom from AF rate of 72.5%. The RECOVER AF^16^ multicentre trial, which examined 106 patients undergoing retreatment, found that 76% remained AF-free at 12 months (67% after a single procedure). These findings support the notion that wavefront pivoting and rotational activity represent meaningful electrophysiological phenomena that contribute to AF maintenance and that targeting these propagation patterns may improve long-term outcomes.

### Low voltage areas

The discordance between LVAs and regions containing CDM propagation patterns challenges the notion that structural remodelling alone determines the functional substrate of AF. This finding aligns with previous studies showing limited efficacy of LVA-guided ablation strategies^10,11^ and suggests that fibrosis may not be the sole determinant of arrhythmogenic substrate in persistent AF.

Several factors may contribute to this discrepancy. Technical challenges exist in LVA measurement, meaning we cannot assume LVAs represent fibrosis; this is evidenced by discordance between EAVM and Magnetic Resonance Imaging based strategies for fibrosis quantification.^31–33^ Even with omnipolar mapping, LVA measurement is impacted by factors such as atrial wall thickness and contact force.^12,24^

Pathophysiologically, electrical remodelling can precede fibrosis, with changes to the action potential, calcium handling, and gap junction remodelling.^34–36^ Multiple previous studies have demonstrated discrepancies between fibrotic regions and CV,^13,37,38^ refractory period,^36,39^ and both observed and simulated AF propagation patterns.^27,40–42^

### Conduction velocity

Reduced atrial CV is a proven predictor of poor ablation outcomes,^12–14,43–46^ and identification of deceleration zones during SR has been used in successful treatment of atrial tachycardias.^47^ However, our study revealed limitations in using CV mapping during paced rhythms to identify regions of functional remodelling as evidenced by CDM propagation patterns.

AF is a variable rate and multidirectional rhythm, both components contributing to heterogeneity in regional CV that is a potential source of functional re-entry.^19,48,49^ To more accurately capture the underlying arrhythmogenic substrate during SR, we combined multiple CL and pacing site protocols to explore the impact of both on CV. Our comprehensive protocol included extrastimulus pacing down to 300 ms; previous work has demonstrated CLs <380 ms can unmask sites of CV slowing and AF initiating spiral propagation not evident in SR,^50^ and that CV decreases even in regions without structural remodelling at CLs <300 ms.^51^ Whilst our results support observations that shorter CLs are associated with slower CVs,^19^ differences in results between our pacing and AF CV protocols highlight limitations of the former in identifying key regions of functional remodelling.

This limitation appears to be primarily mediated through the impact of pacing itself on CV. Pacing site may influence CV due to anisotropic fibre orientation. However, despite CS pacing providing the most physiological course for left atrial activation, it did not display the greatest CV. Instead, we observed CV to be slowest around the site of pacing, or Bachmann’s bundle in the case of RAA pacing. Therefore, it appears CV is more influenced by wavefront curvature as propagation emanates from pacing sites,^52^ an observation noted in previous studies.^19^ Consequently, even multi-CL, multi-site pacing protocols face inherent challenges in accurately representing the underlying substrate, which may explain the lack of correlation with CDM propagation patterns.

The observed relationship between slow CV during AF and the localization of pivoting and rotational propagation sites can be elucidated through the source-sink relationship of the propagating wavefront; this explains how curvature of a wavefront influences CV. Where the leading edge of a planar wavefront couples to an equivalent number of cells ahead of it, a convex wavefront couples to a greater number of cells ahead of it. Consequently, for a consistent source size, the sink is more substantial in a convex wavefront and CV slower. This explanation is well established, being the basis of spiral wave theory.^53–55^

### Consistent anatomical regions

An intriguing observation from our study is the consistent localization of CDM propagation patterns to two specific anatomical regions across patients, including in both induced and spontaneous AF: the anteroseptal and inferoposterior walls. Multiple previous studies have also demonstrated CDM propagation patterns in these areas, reinforcing their potential significance in AF dynamics.^16,25,27,38^

Several factors may contribute to their involvement, including anisotropic fibre alignment due to the septopulmonary bundle,^14,56,57^ embryological relation with the PV,^8^ and mechanical pressure from left atrium-adjacent structures promoting remodelling.^14,58^ Furthermore, the average body mass index of our cohort was 30.8 kg/m^2^; obesity has previously been associated with increased adipose tissue adjacent to the posterior left atrium and LVAs within this region.^59^

While limited by sample size and study design, our clinical outcomes provide additional context. The interpretation of recurrence rates should consider that it was defined based on clinical follow-up rather than systematic monitoring. There was no pre-specified follow-up protocol and only 12-month recurrence was assessed, whereas AF burden is increasingly recognized as a clinically meaningful endpoint.^2,60^

Nevertheless, 11 patients (50%) underwent some degree of ablation in these areas, with two of the three documented recurrences occurring in this cohort. Only one patient in this study underwent ablation of both the anteroseptal and inferoposterior regions, and this patient did not experience recurrence. These findings suggest that a comprehensive ablation strategy incorporating all these regions may be necessary. This could, in part, explain the negative outcomes of existing empirical approaches, such as posterior wall isolation,^8^ where ablation lesions neither extend inferiorly enough nor include the anteroseptal wall.

Whilst incomplete identification of arrhythmogenic substrate is an important mechanism of AF recurrence, it is inherently multifactorial, involving additional factors such as progressive substrate remodelling and limitations in ablation lesion durability. Fully elucidating the mechanisms of AF recurrence would ideally require repeat mapping post-recurrence, which was beyond the scope of this study.

### Clinical implications

Our findings have important clinical implications. While highly individualized mapping approaches using advanced technologies like CDM have shown promise,^15,16,22^ limited availability restricts widespread adoption; notably, the AcQMap system is currently not clinically available.

Our observation that CV mapping during paced rhythms fails to reliably identify regions containing CDM propagation patterns highlights the limitations of assessing functional substrate outside the context of fibrillation. However, CV assessment using EAVM during AF is technically challenging. Electrograms are often complex, fractionated, and low-voltage, with multiple wavefronts that can collide or take re-entrant paths; this leads to difficulties in determining LAT, with subsequent errors in CV estimates.^33,61^

These limitations emphasise the need for more practical methods to identify critical substrate regions. The identification of consistent anatomical targets offers the potential for a standardized, empirical ablation strategy that could be implemented across centres without the need for specialized equipment. This approach aligns well with emerging pulsed-field ablation technologies, which offer improved safety profiles and efficient lesion creation over broader anatomical regions.

While our study provides a strong foundation for this hypothesis, independent validation in larger studies is necessary. Future randomized controlled trials should evaluate whether empirical ablation of these anatomical regions, in addition to PVI, leads to improved procedural outcomes and long-term arrhythmia-free survival in patients with persistent AF.

### Limitations

The benefit of targeting propagation patterns identified through CDM has been demonstrated in prospective single-arm cohort studies,^15,16,22^ but not randomized controlled trials. Charge density mapping derived electrograms have been validated against contact mapping during both SR and AF,^21^ and our study highlights important differences between propagation abnormalities identified using CDM and alternative functional and structural approaches to identifying regions of negative atrial remodelling. Not all mapping protocols were performed in all patients, potentially impacting population-wide measures. The use of general anaesthesia in all procedures may have influenced autonomic tone, potentially affecting mapping results. Some AF episodes were spontaneous while others were induced, and some patients were on amiodarone; we have presented data based on subpopulation analyses where appropriate, and individual cases are included in Supplementary material online, *Figure S5*. Whilst the right atrium is known to play a role in the maintenance of AF, this study was limited to the left atrium.

## Conclusion

Our study demonstrates that conventional mapping techniques during paced rhythms do not fully capture the arrhythmogenic substrate of persistent AF. However, we identified two consistent anatomical regions where pivoting and rotational propagation patterns frequently occur during AF across patients, suggesting a predisposition to electrophysiological abnormalities. As these regions can be identified without specialized mapping technologies, further investigation is warranted to determine their potential role in empirical ablation strategies for persistent AF.

## Supplementary Material

euaf048_Supplementary_Data

## Data Availability

The data underlying this article will be shared on reasonable request to the corresponding author.

## References

[1] Haissaguerre M, Jais P, Shah D, Takahashi A, Hocini M, Quiniou G et al Spontaneous initiation of atrial fibrillation by ectopic impulses originating in the pulmonary veins. N Engl J Med 1998;339:659–66. 9725923 10.1056/NEJM199809033391003

[2] Tzeis S, Gerstenfeld EP, Kalman J, Saad EB, Sepehri Shamloo A, Andrade JG et al 2024 European Heart Rhythm Association/Heart Rhythm Society/Asia Pacific Heart Rhythm Society/Latin American Heart Rhythm Society expert consensus statement on catheter and surgical ablation of atrial fibrillation. Europace 2024;26:euae043. 10.1093/europace/euae043 PMC11000153 38587017

[3] Hindricks G, Potpara T, Dagres N, Bax JJ, Boriani G, Dan GA et al 2020 ESC guidelines for the diagnosis and management of atrial fibrillation developed in collaboration with the European Association for Cardio-Thoracic Surgery (EACTS). Eur Heart J 2021;42:373–498. 32860505 10.1093/eurheartj/ehaa612

[4] Winkle RA, Mead RH, Engel G, Salcedo J, Brodt C, Barberini P et al Very long term outcomes of atrial fibrillation ablation. Heart Rhythm 2023;20:680–8. 36764350 10.1016/j.hrthm.2023.02.002

[5] Clarnette JA, Brooks AG, Mahajan R, Elliott AD, Twomey DJ, Pathak RK et al Outcomes of persistent and long-standing persistent atrial fibrillation ablation: a systematic review and meta-analysis. Europace 2018;20:f366–76. 29267853 10.1093/europace/eux297

[6] Boersma L, Andrade JG, Betts T, Duytschaever M, Pürerfellner H, Santoro F et al Progress in atrial fibrillation ablation during 25 years of Europace journal. Europace 2023;25:1–14. 37622592 10.1093/europace/euad244 PMC10451004

[7] Verma A, Jiang C, Betts TR, Chen J, Deisenhofer I, Mantovan R et al Approaches to catheter ablation for persistent atrial fibrillation. N Engl J Med 2015;372:1812–22. 25946280 10.1056/NEJMoa1408288

[8] Kistler PM, Chieng D, Sugumar H, Ling LH, Segan L, Azzopardi S et al Effect of catheter ablation using pulmonary vein isolation with vs without posterior left atrial wall isolation on atrial arrhythmia recurrence in patients with persistent atrial fibrillation: the CAPLA randomized clinical trial. JAMA 2023;329:127–35. 36625809 10.1001/jama.2022.23722 PMC9856612

[9] Inoue K, Hikoso S, Masuda M, Furukawa Y, Hirata A, Egami Y et al Pulmonary vein isolation alone vs. More extensive ablation with defragmentation and linear ablation of persistent atrial fibrillation: the EARNEST-PVI trial. Europace 2021;23:565–74. 33200213 10.1093/europace/euaa293

[10] Huo Y, Gaspar T, Schönbauer R, Wójcik M, Fiedler L, Roithinger FX et al Low-voltage myocardium-guided ablation trial of persistent atrial fibrillation. NEJM Evid 2022;1:EVIDoa2200141. 38319851 10.1056/EVIDoa2200141

[11] Mohanty S, Mohanty P, Di Biase L, Trivedi C, Morris EH, Gianni C et al Long-term follow-up of patients with paroxysmal atrial fibrillation and severe left atrial scarring: comparison between pulmonary vein antrum isolation only or pulmonary vein isolation combined with either scar homogenization or trigger ablation. Europace 2017;19:1790–7. 28039211 10.1093/europace/euw338

[12] Okubo Y, Oguri N, Sakai T, Uotani Y, Furutani M, Miyamoto S et al Conduction velocity mapping in atrial fibrillation using omnipolar technology. Pacing Clin Electrophysiol 2024;47:19–27. 38041418 10.1111/pace.14899

[13] Kuo MJ, Ton ANK, Lo LW, Lin YJ, Chang SL, Hu YF et al Abnormal conduction zone detected by isochronal late activation mapping accurately identifies the potential atrial substrate and predicts the atrial fibrillation ablation outcome after pulmonary vein isolation. Circ Arrhythm Electrophysiol 2023;16:E011149. 36688314 10.1161/CIRCEP.122.011149

[14] Ohguchi S, Inden Y, Yanagisawa S, Fujita R, Yasuda K, Katagiri K et al Regional left atrial conduction velocity in the anterior wall is associated with clinical recurrence of atrial fibrillation after catheter ablation: efficacy in combination with the ipsilateral low voltage area. BMC Cardiovasc Disord 2022;22:457. 36319975 10.1186/s12872-022-02881-6 PMC9628089

[15] Willems S, Verma A, Betts TR, Murray S, Neuzil P, Ince H et al Targeting nonpulmonary vein sources in persistent atrial fibrillation identified by noncontact charge density mapping: UNCOVER AF trial. Circ Arrhythm Electrophysiol 2019;12:1–12. 10.1161/CIRCEP.119.007233 31242746

[16] Betts TR, Good WW, Melki L, Metzner A, Grace A, Verma A et al Treatment of pathophysiologic propagation outside of the pulmonary veins in retreatment of atrial fibrillation patients: RECOVER AF study. Europace 2023;25:euad097. 37072340 10.1093/europace/euad097 PMC10228624

[17] Kawaji T, Hojo S, Kushiyama A, Nakatsuma K, Kaneda K, Kato M et al Optimal cutoff value of bipolar low-voltage in electroanatomic voltage mapping during atrial fibrillation rhythm. Pacing Clin Electrophysiol 2019;42:663–9. 30873619 10.1111/pace.13661

[18] Williams SE, Linton N, O’Neill L, Harrison J, Whitaker J, Mukherjee R et al The effect of activation rate on left atrial bipolar voltage in patients with paroxysmal atrial fibrillation. J Cardiovasc Electrophysiol 2017;28:1028–36. 28639747 10.1111/jce.13282 PMC5639376

[19] Wong GR, Nalliah CJ, Lee G, Voskoboinik A, Prabhu S, Parameswaran R et al Dynamic atrial substrate during high-density mapping of paroxysmal and persistent AF: implications for substrate ablation. JACC Clin Electrophysiol 2019;5:1265–77. 31753431 10.1016/j.jacep.2019.06.002

[20] Grace A, Willems S, Meyer C, Verma A, Heck P, Zhu M et al High-resolution noncontact charge-density mapping of endocardial activation. JCI Insight 2019;4:0–19. 10.1172/jci.insight.126422 PMC6483005 30895945

[21] Shi R, Parikh P, Chen Z, Angel N, Norman M, Hussain W et al Validation of dipole density mapping during atrial fibrillation and Sinus rhythm in human left atrium. JACC Clin Electrophysiol 2020;6:171–81. 32081219 10.1016/j.jacep.2019.09.012

[22] Shi R, Chen Z, Pope MTB, Zaman JAB, Debney M, Marinelli A et al Individualized ablation strategy to treat persistent atrial fibrillation: core-to-boundary approach guided by charge-density mapping. Heart Rhythm 2021;18:862–70. 33610744 10.1016/j.hrthm.2021.02.014

[23] Pope MTB, Leo M, Briosa e Gala A, Betts TR. Clinical utility of non-contact charge density ‘SuperMap’ algorithm for the mapping and ablation of organized atrial arrhythmias. Europace 2022;24:747–54. 34871398 10.1093/europace/euab271 PMC9071092

[24] Van Schie MS, Kharbanda RK, Houck CA, Lanters EAH, Taverne YJHJ, Bogers AJJC et al Identification of low-voltage areas: a unipolar, bipolar, and omnipolar perspective. Circ Arrhythm Electrophysiol 2021;14:E009912. 34143644 10.1161/CIRCEP.121.009912 PMC8294660

[25] Dang L, Angel N, Zhu M, Vesin JM, Scharf C. Correlation between conduction velocity and frequency analysis in patients with atrial fibrillation using high-density charge mapping. Med Biol Eng Comput 2022;60:3081–90. 36065071 10.1007/s11517-022-02659-0

[26] Mickelsen SR, Angel N, Shah P, Shi X, Chou D. B-PO05–085 regional conduction velocity measurements: comparing contact and non-contact activation techniques. Heart Rhythm 2021;18:S406.

[27] Pope MTB, Kuklik P, Briosa e Gala A, Leo M, Mahmoudi M, Paisey J et al Spatial and temporal variability of rotational, focal, and irregular activity: practical implications for mapping of atrial fibrillation. J Cardiovasc Electrophysiol 2021;32:2393–403. 34260134 10.1111/jce.15170 PMC9290790

[28] Cates J, Elhabian S, Whitaker R. ShapeWorks: particle-based shape correspondence and visualization software. In: Statistical Shape and Deformation Analysis: Methods, Implementation and Applications. Elsevier Inc; 2017:p257–98.

[29] Nattel S, Xiong F, Aguilar M. Demystifying rotors and their place in clinical translation of atrial fibrillation mechanisms. Nat Rev Cardiol 2017;14:509–20. 28383023 10.1038/nrcardio.2017.37

[30] Xu CH, Xiong F, Jiang WF, Liu X, Liu T, Qin M. Rotor mechanism and its mapping in atrial fibrillation. Europace 2023;25:783–92. 36734272 10.1093/europace/euad002 PMC10062333

[31] Nairn D, Eichenlaub M, Müller-Edenborn B, Huang T, Lehrmann H, Nagel C et al Differences in atrial substrate localization using LGE-MRI, electrogram voltage and conduction velocity—a cohort study using a consistent anatomical reference frame in patients with persistent atrial fibrillation. Europace 2023;25:euad278. 37713626 10.1093/europace/euad278 PMC10533207

[32] Eichenlaub M, Mueller-Edenborn B, Minners J, Ventura F, Forcada RMI, Colomer BR et al Comparison of various late gadolinium enhancement magnetic resonance imaging methods with high-definition voltage and activation mapping for detection of atrial cardiomyopathy. Europace 2022;24:1102–11. 35298612 10.1093/europace/euac010

[33] Chen J, Arentz T, Cochet H, Müller-Edenborn B, Kim S, Moreno-Weidmann Z et al Extent and spatial distribution of left atrial arrhythmogenic sites, late gadolinium enhancement at magnetic resonance imaging, and low-voltage areas in patients with persistent atrial fibrillation: comparison of imaging vs. Electrical parameters of fibrosis and arrhythmogenesis. Europace 2019;21:1484–93. 31280323 10.1093/europace/euz159

[34] Jansen HJ, Bohne LJ, Gillis AM, Rose RA. Atrial remodeling and atrial fibrillation in acquired forms of cardiovascular disease. Heart Rhythm O2 2020;1:147–59. 34113869 10.1016/j.hroo.2020.05.002 PMC8183954

[35] Coveney S, Cantwell C, Roney C. Atrial conduction velocity mapping: clinical tools, algorithms and approaches for understanding the arrhythmogenic substrate. Med Biol Eng Comput 2022;60:2463–78. 35867323 10.1007/s11517-022-02621-0 PMC9365755

[36] Williams SE, Linton NWF, Harrison J, Chubb H, Whitaker J, Gill J et al Intra-atrial conduction delay revealed by multisite incremental atrial pacing is an independent marker of remodeling in human atrial fibrillation. JACC Clin Electrophysiol 2017;3:1006–17. 28966986 10.1016/j.jacep.2017.02.012 PMC5612260

[37] Silva Garcia E, Lobo-Torres I, Fernández-Armenta J, Penela D, Fernandez-Garcia M, Gomez-Lopez A et al Functional mapping to reveal slow conduction and substrate progression in atrial fibrillation. Europace 2023;25:euad246. 10.1093/europace/euad246 PMC10644200 37961921

[38] Zahid S, Malik T, Peterson C, Tarabanis C, Dai M, Katz M et al Conduction velocity is reduced in the posterior wall of hypertrophic cardiomyopathy patients with normal bipolar voltage undergoing ablation for paroxysmal atrial fibrillation. J Interv Card Electrophysiol 2024;67:203–10. 36952090 10.1007/s10840-023-01533-9

[39] Li B, Luo F, Luo X, Li B, Qi L, Zhang D et al Effects of atrial fibrosis induced by mitral regurgitation on atrial electrophysiology and susceptibility to atrial fibrillation in pigs. Cardiovasc Pathol 2019;40:32–40. 30836303 10.1016/j.carpath.2019.01.006

[40] Lee JMS, Nelson TA, Clayton RH, Kelland NF. Characterization of persistent atrial fibrillation with non-contact charge density mapping and relationship to voltage. J Arrhythm 2022;38:77–85. 35222753 10.1002/joa3.12661 PMC8851595

[41] Chierchia GB, Sieira J, Vanderper A, Osorio TG, Bala G, Stroker E et al Substrate mapping of the left atrium in persistent atrial fibrillation: spatial correlation of localized complex conduction patterns in global charge-density maps to low-voltage areas in 3D contact bipolar voltage maps. J Interv Card Electrophysiol 2021;62:539–47. 33420713 10.1007/s10840-020-00926-4 PMC8645534

[42] Boyle PM, Hakim JB, Zahid S, Franceschi WH, Murphy MJ, Vigmond EJ et al Comparing reentrant drivers predicted by image-based computational modeling and mapped by electrocardiographic imaging in persistent atrial fibrillation. Front Physiol 2018;9:1–12. 29725307 10.3389/fphys.2018.00414 PMC5917348

[43] Jadidi A, Müller-Edenborn B, Chen J, Keyl C, Weber R, Allgeier J et al The duration of the amplified Sinus-P-wave identifies presence of left atrial low voltage substrate and predicts outcome after pulmonary vein isolation in patients with persistent atrial fibrillation. JACC Clin Electrophysiol 2018;4:531–43. 30067494 10.1016/j.jacep.2017.12.001

[44] Invers-Rubio E, Hernández-Romero I, Reventos-Presmanes J, Ferro E, Guichard J-B, Regany-Closa M et al Regional conduction velocities determined by non-invasive mapping are associated with arrhythmia-free survival after atrial fibrillation ablation. Heart Rhythm 2024;9:1570–80. 10.1016/j.hrthm.2024.04.063 38636930

[45] Qi D, Guan X, Liu X, Liu L, Liu Z, Zhang J. Slow conduction velocity predicts atrial fibrillation recurrence after radiofrequency ablation. J Cardiovasc Electrophysiol 2024;35:461–8. 38282308 10.1111/jce.16193

[46] Kurata N, Masuda M, Kanda T, Asai M, Iida O, Okamoto S et al Slow whole left atrial conduction velocity after pulmonary vein isolation predicts atrial fibrillation recurrence. J Cardiovasc Electrophysiol 2020;31:1942–9. 32445427 10.1111/jce.14582

[47] Yorgun H, Çöteli C, Kılıç GS, Sezenöz B, Dural M, Ateş AH et al Functional substrate mapping characteristics during sinus rhythm predicts critical isthmus of reentrant atrial tachycardia. J Cardiovasc Electrophysiol 2023;34:1539–48. 37269230 10.1111/jce.15961

[48] Markides V, Schilling RJ, Ho SY, Chow AWC, Davies DW, Peters NS. Characterization of left atrial activation in the intact human heart. Circulation 2003;107:733–9. 12578877 10.1161/01.cir.0000048140.31785.02

[49] Wong CX, John B, Brooks AG, Chandy ST, Kuklik P, Lau DH et al Direction-dependent conduction abnormalities in the chronically stretched atria. Europace 2012;14:954–61. 22308090 10.1093/europace/eur428

[50] Schricker AA, Lalani GG, Krummen DE, Rappel WJ, Narayan SM. Human atrial fibrillation initiates via organized rather than disorganized mechanisms. Circ Arrhythm Electrophysiol 2014;7:816–24. 25217042 10.1161/CIRCEP.113.001289 PMC4206587

[51] Honarbakhsh S, Schilling RJ, Orini M, Providencia R, Keating E, Finlay M et al Structural remodeling and conduction velocity dynamics in the human left atrium: relationship with reentrant mechanisms sustaining atrial fibrillation. Heart Rhythm 2019;16:18–25. 30026014 10.1016/j.hrthm.2018.07.019 PMC6317307

[52] Padilla JR, Anderson RD, Joens C, Masse S, Bhaskaran A, Niri A et al Orientation of conduction velocity vectors on cardiac mapping surfaces. Europace 2023;25:1172–82. 36609707 10.1093/europace/euac259 PMC10062359

[53] Roney CH, Wit AL, Peters NS. Challenges associated with interpreting mechanisms of AF. Arrhythm Electrophysiol Rev 2019;8:273–84. 10.15420/aer.2019.08 PMC7358959 32685158

[54] Comtois P, Kneller J, Nattel S. Of circles and spirals: bridging the gap between the leading circle and spiral wave concepts of cardiac reentry. Europace 2005;7:10–20. 16102499 10.1016/j.eupc.2005.05.011

[55] Pandit SV, Jalife J. Rotors and the dynamics of cardiac fibrillation. Circ Res 2013;112:849–62. 23449547 10.1161/CIRCRESAHA.111.300158 PMC3650644

[56] Kishima H, Mine T, Fukuhara E, Takahashi S, Ishihara M. Is the abnormal conduction zone of the left atrium a precursor to a low voltage area in patients with atrial fibrillation? J Cardiovasc Electrophysiol 2020;31:2874–82. 32936499 10.1111/jce.14744

[57] Papez JW . Heart musculature of the atria. Am J Anat 1920;27:255–85.

[58] Nakahara S, Yamaguchi T, Hori Y, Anjo N, Hayashi A, Kobayashi S et al Spatial relation between left atrial anatomical contact areas and circular activation in persistent atrial fibrillation. J Cardiovasc Electrophysiol 2016;27:515–23. 26725874 10.1111/jce.12907

[59] Mahajan R, Nelson A, Pathak RK, Middeldorp ME, Wong CX, Twomey DJ et al Electroanatomical remodeling of the atria in obesity: impact of adjacent epicardial fat. JACC Clin Electrophysiol 2018;4:1529–40. 30573116 10.1016/j.jacep.2018.08.014

[60] Becher N, Metzner A, Toennis T, Kirchhof P, Schnabel RB. Atrial fibrillation burden: a new outcome predictor and therapeutic target. Eur Heart J 2024;45:2824–38. 38953776 10.1093/eurheartj/ehae373 PMC11328870

[61] Groot NMS, Shah D, Boyle PM, Anter E, Clifford GD, Deisenhofer I et al Critical appraisal of technologies to assess electrical activity during atrial fibrillation: a position paper from the European heart rhythm association and European Society of Cardiology Working Group on eCardiology in collaboration with the Heart Rhythm Society, Asia Pacific Heart Rhythm Society, Latin American Heart Rhythm Society and Computing in Cardiology. Europace 2022;24:313–30. 34878119 10.1093/europace/euab254 PMC11636570

