# Supplementary material for: Identifying extra pulmonary vein targets for persistent atrial fibrillation ablation: bridging advanced and conventional mapping techniques
Source: Europace. 2025 Mar 12;27(4):euaf048. doi: 10.1093/europace/euaf048 (PMC11953006; doi:10.1093/europace/euaf048)
Supplement: euaf048_Supplementary_Data [file euaf048_supplementary_data.pdf]

Identifying Extra Pulmonary Vein Targets for Persistent Atrial Fibrillation Ablation: Bridging Advanced and Conventional Mapping Techniques

Supplementary Materials

Page	Element
2	*Table S1*. **Detailed clinical characteristics of the study population**
3	*Table S2*. **Accuracy of registrations with template geometry**
4	*Table S3.* **Individual AF recurrence outcomes and locations of additional ablation lesions**
5	*Figure S1.* **Individual registrations with template geometry**
6	*Figure S2.* **Non-amiodarone taking population spatial distributions of CDM propagation patterns**
7	*Figure S3.* **Non-amiodarone taking population spatial distributions of voltage**
8	*Figure S4.* **Non-amiodarone taking population spatial distributions of conduction velocity**
9 – 11	*Figure S5.* **Individual spatial distributions**
12	**Supplementary Figure legends**

Table S1. **Detailed clinical characteristics of study population.**

**Characteristics**	**Distribution**
Medical history, n (%)	
Coronary artery disease	2 (9)
Congestive heart failure	9 (41)
Valvular heart disease	1 (5)
Hypertension	12 (55)
Diabetes mellitus	1 (5)
Asthma / chronic obstructive pulmonary disease	3 (14)
Obstructive sleep apnoea	1 (5)
Chronic kidney disease	3 (14)
Social history, n (%)	
Smoker	5 (22)
>14 units alcohol per week	2 (9)

Table S2. **Accuracy of registrations with template geometry.**

**Case**	**Distance to template, mm, mean ± SD**	
	CDM	EAVM
1	3.9 ± 3.0	3.2 ± 3.0
2	3.1 ± 2.0	4.9 ± 3.5
3	2.1 ± 1.0	3.6 ± 2.5
4	1.8 ± 0.9	
5	3.3 ± 1.4	3.0 ± 2.2
6	2.3 ± 1.5	3.8 ± 2.2
7	2.7 ± 1.8	4.5 ± 3.0
8	3.8 ± 2.1	3.4 ± 2.3
9	1.4 ± 0.7	3.1 ± 2.3
10	2.6 ± 1.6	4.6 ± 2.7
11	2.0 ± 1.0	
12	1.5 ± 0.8	4.9 ± 3.0
13	2.3 ± 1.6	4.7 ± 3.2
14	1.6 ± 1.1	3.2 ± 2.2
15	2.6 ± 1.7	3.6 ± 2.7
16	3.2 ± 2.5	3.7 ± 2.7
17	2.2 ± 1.6	4.1 ± 2.6
18	3.6 ± 2.3	3.7 ± 2.6
19	3.5 ± 2.0	4.6 ± 3.2
20	3.0 ± 1.8	4.0 ± 3.0
21	4.2 ± 2.2	4.3 ± 2.8
22	2.4 ± 1.8	3.5 ± 2.4

CDM: charge density mapping; EAVM: electroanatomic voltage mapping; SD: standard deviation.

Table S3. **Individual AF recurrence outcomes and locations of additional ablation lesions.**

**Case**	**AF recurrence**	***Locations of additional ablation lesions***					
		Anterior	Septal	*Posterior*	Inferior	Roof	Lateral
1	No	**Yes**	No	**Yes**	No	No	No
2	No	**Yes**	No	No	No	Yes	No
3	No	**Yes**	**Yes**	**Yes**	No	No	No
4	**Yes**	**Yes**	No	**Yes**	**Yes**	No	No
5	No	**Yes**	No	**Yes**	No	No	No
6	No	No	No	**Yes**	No	No	No
7	No	**Yes**	No	No	No	**Yes**	No
8	No	**Yes**	No	**Yes**	No	**Yes**	No
9	No	No	No	**Yes**	No	No	No
10	No	No	No	No	No	No	No
11	**Yes**	**Yes**	No	No	**Yes**	**Yes**	No
12	No	No	No	No	No	No	No
13	No	No	No	No	No	No	No
14	No	No	No	No	No	No	No
15	No	No	No	No	No	No	No
16	No	No	No	No	No	No	No
17	No	No	No	**Yes**	No	No	No
18	No	**Yes**	No	No	No	No	No
19	No	No	No	No	No	No	No
20	**Yes**	No	No	No	**Yes**	No	No
21	No	No	No	No	No	No	No
22	No	No	No	No	No	No	No

AF: atrial fibrillation; CDM: charge density mapping.

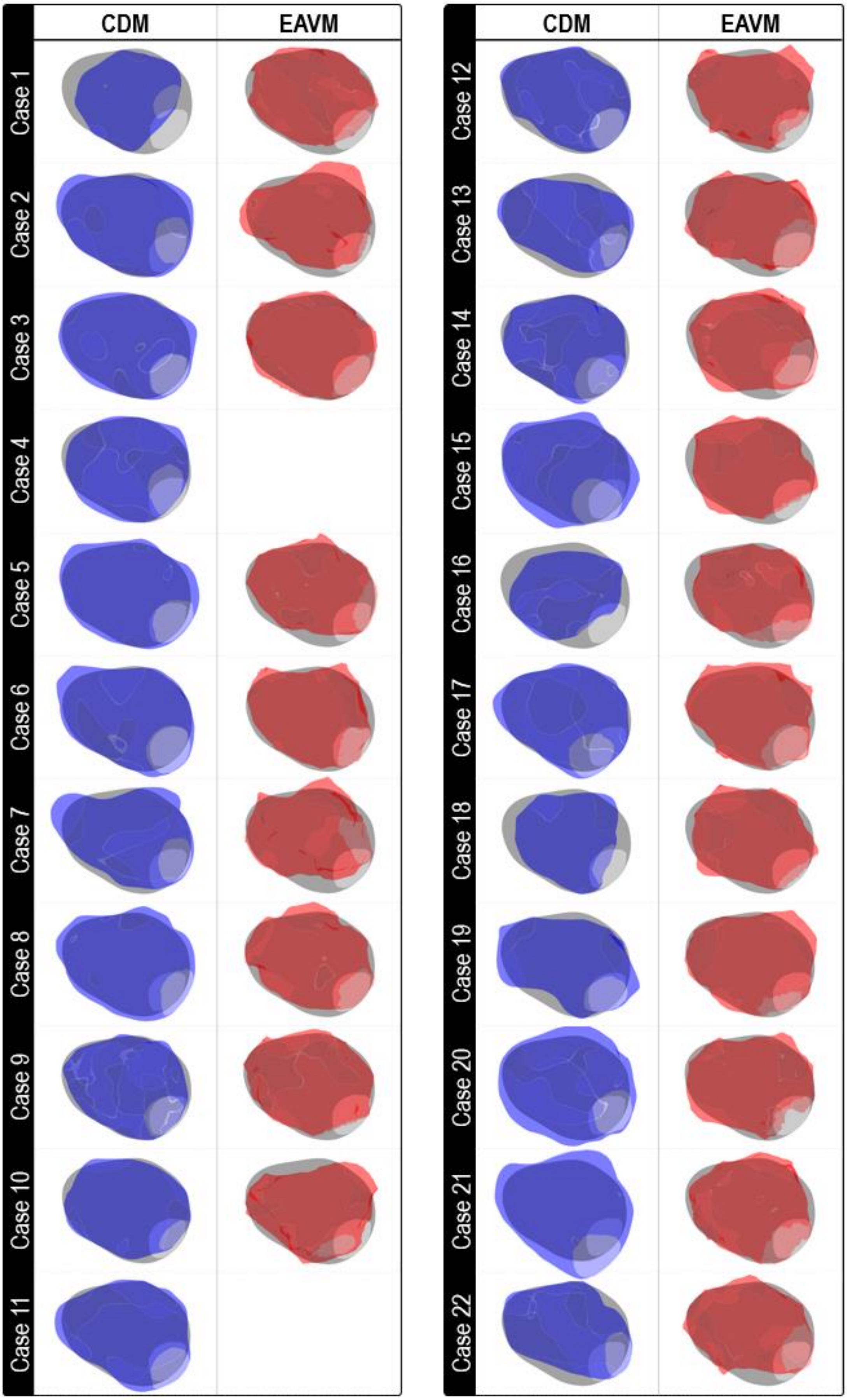

Figure S1. **Individual registrations with template geometry.**

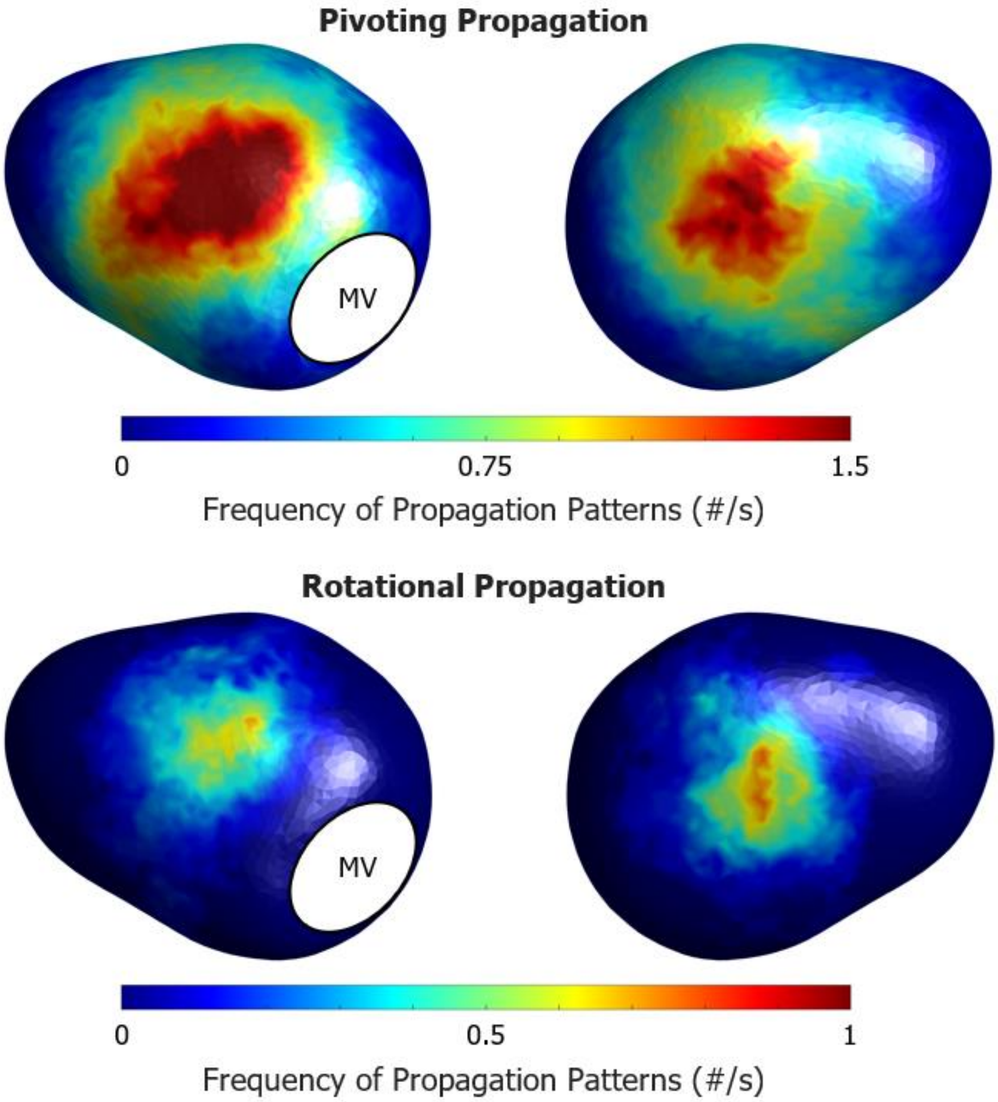

Figure S2. **Non-amiodarone taking population spatial distributions of CDM propagation patterns.**

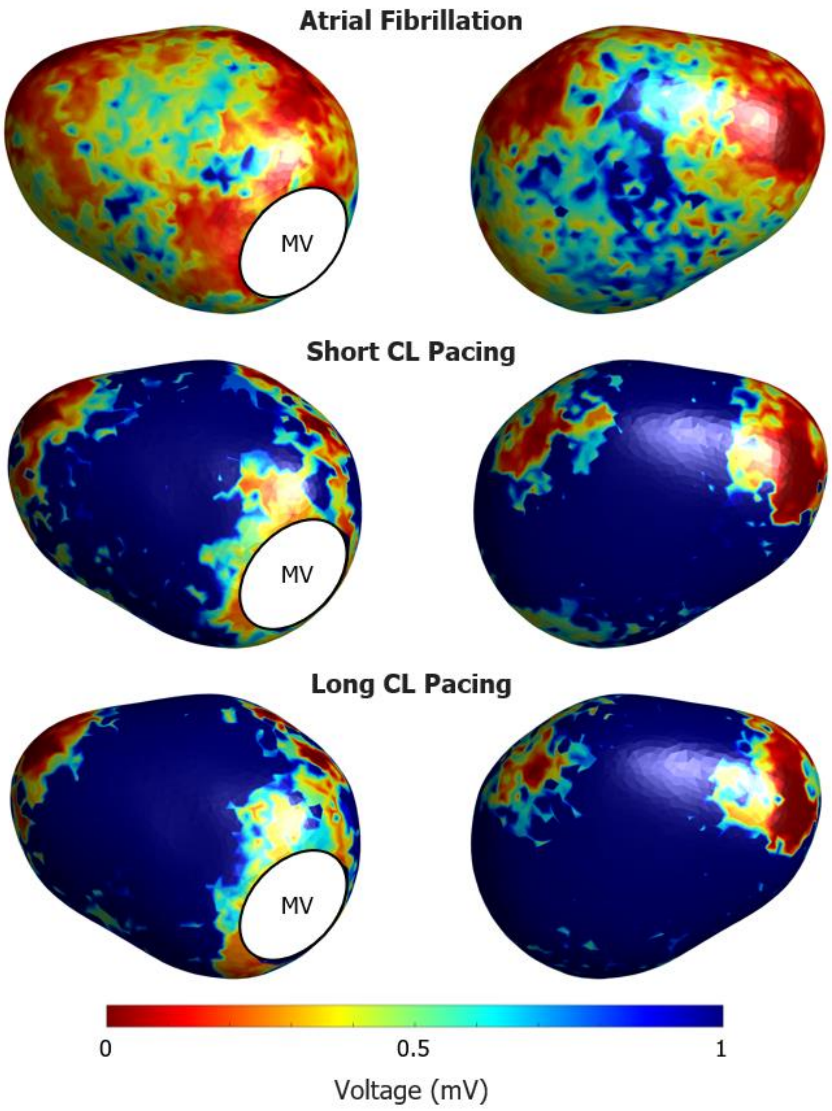

Figure S3. **Non-amiodarone taking population spatial distributions of voltage.**

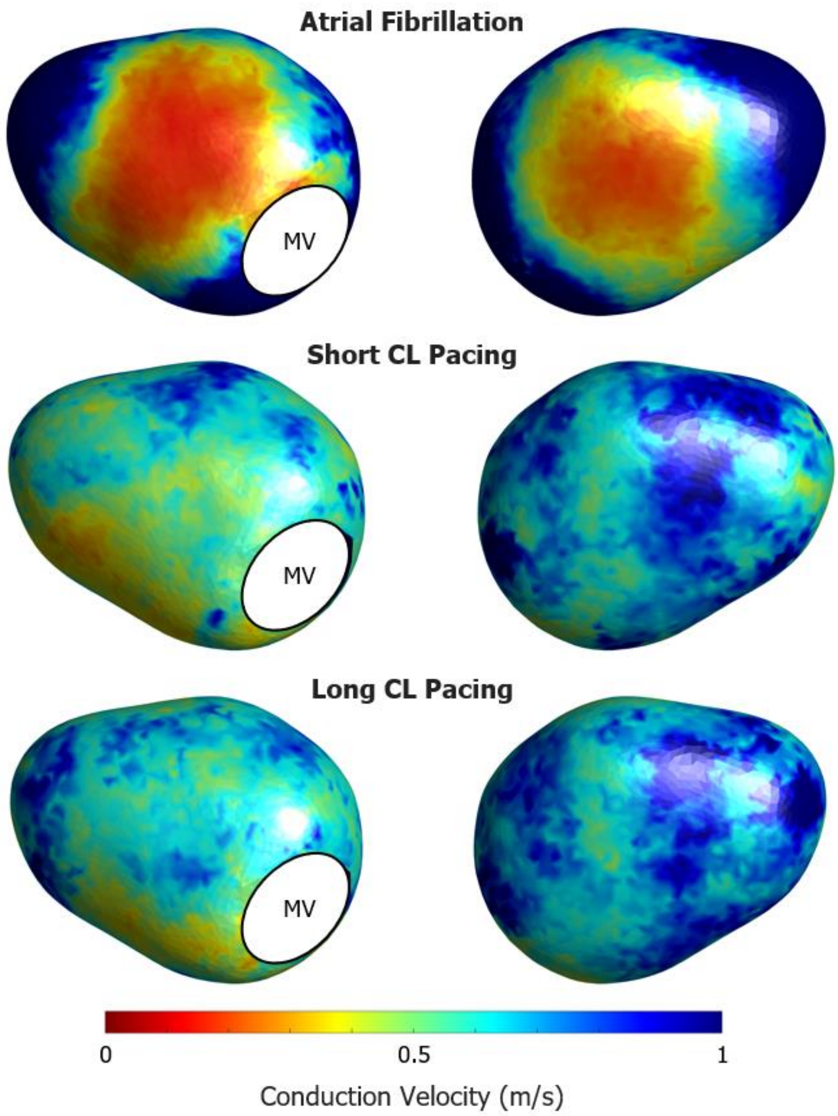

Figure S4. **Non-amiodarone taking population spatial distributions of conduction velocity.**

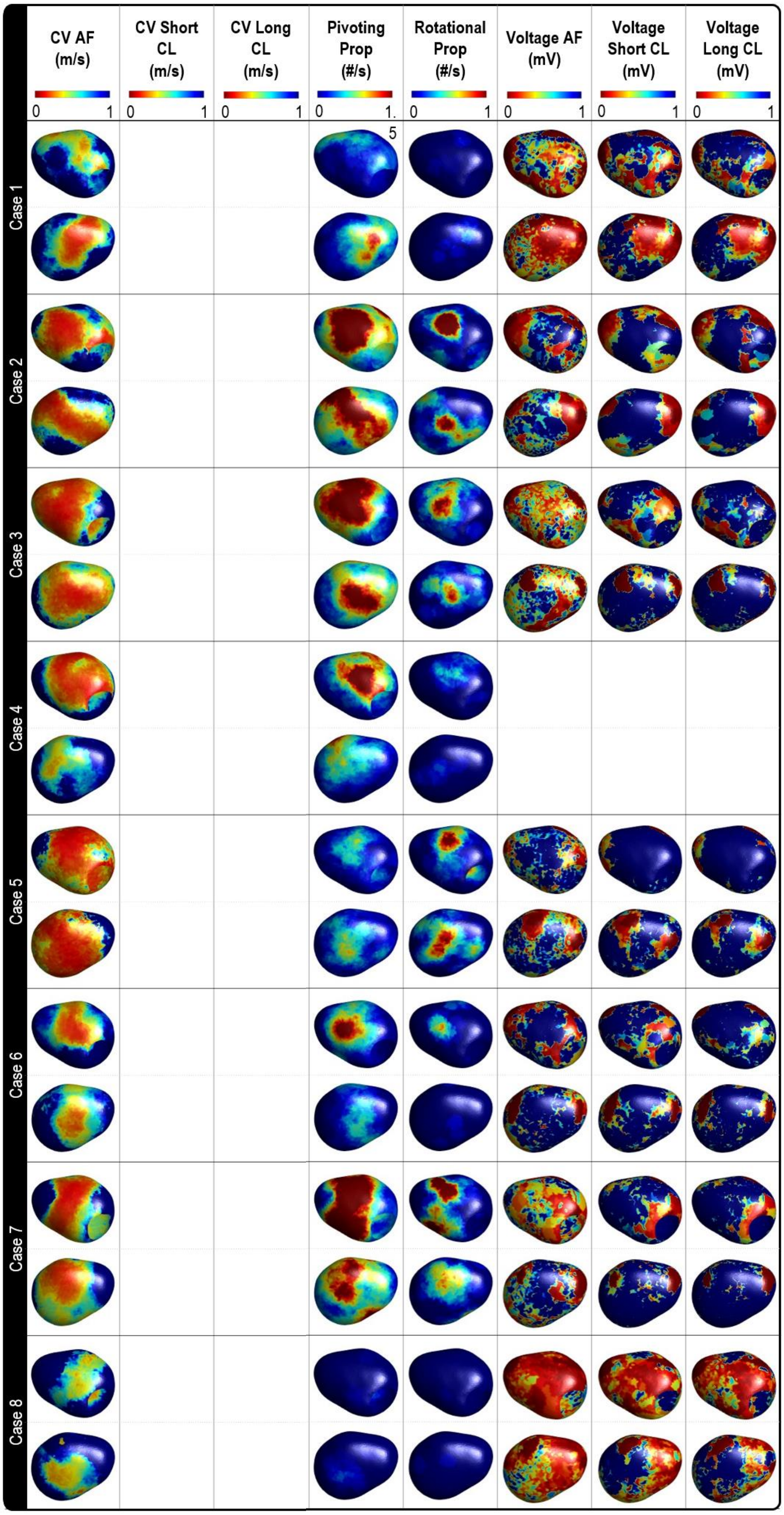
CV AF (m/s)
CV Short CL (m/s)
CV Long CL (m/s)
Pivoting Prop (#/s)
Rotational Prop (#/s)
Voltage AF (mV)
Voltage Short CL (mV)
Voltage Long CL (mV)
0 1
0 1.5
Case 1
Case 2
Case 3
Case 4
Case 5
Case 6
Case 7
Case 8

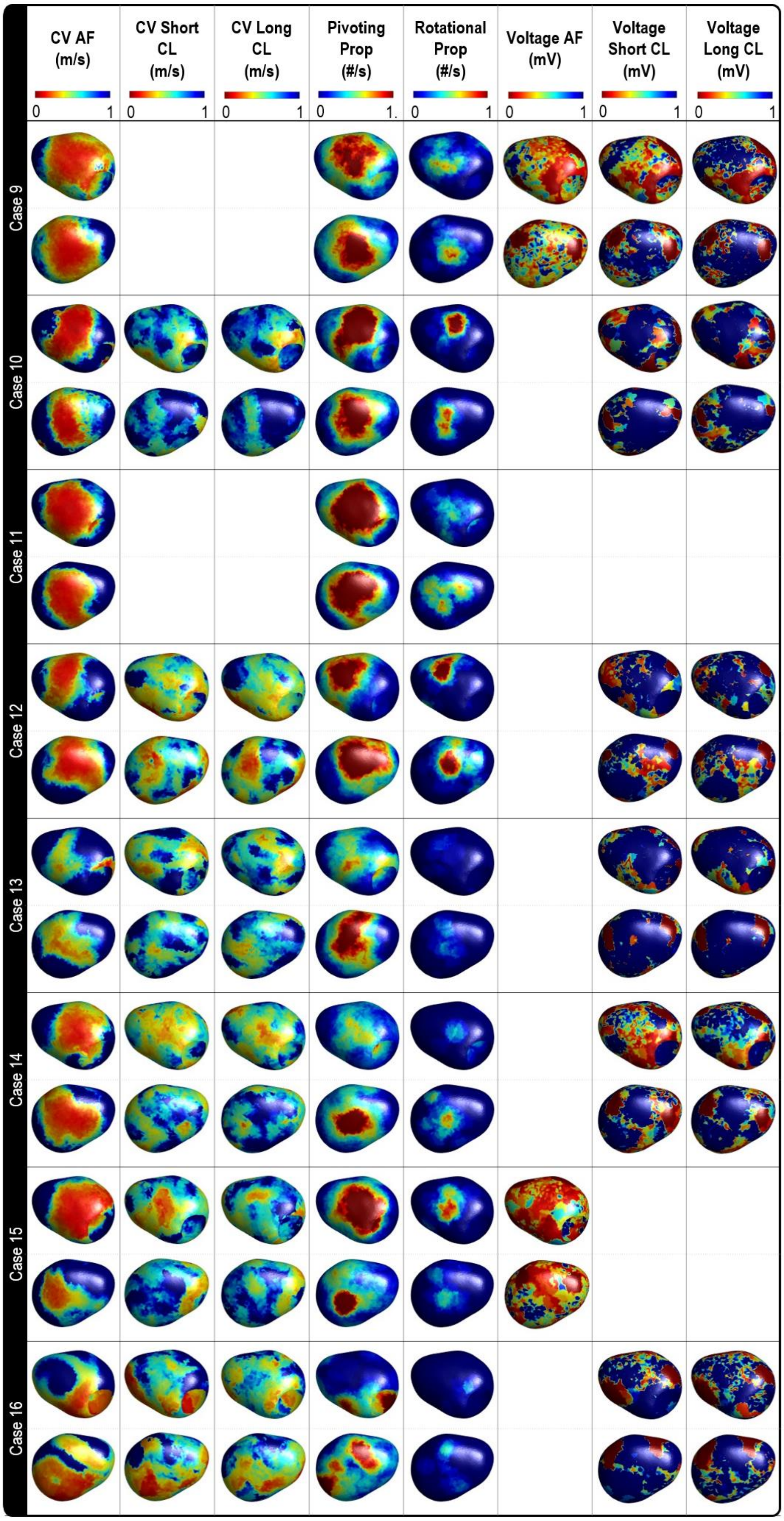
CV AF (m/s)
CV Short CL (m/s)
CV Long CL (m/s)
Pivoting Prop (#/s)
Rotational Prop (#/s)
Voltage AF (mV)
Voltage Short CL (mV)
Voltage Long CL (mV)
0 1
Case 9
Case 10
Case 11
Case 12
Case 13
Case 14
Case 15
Case 16

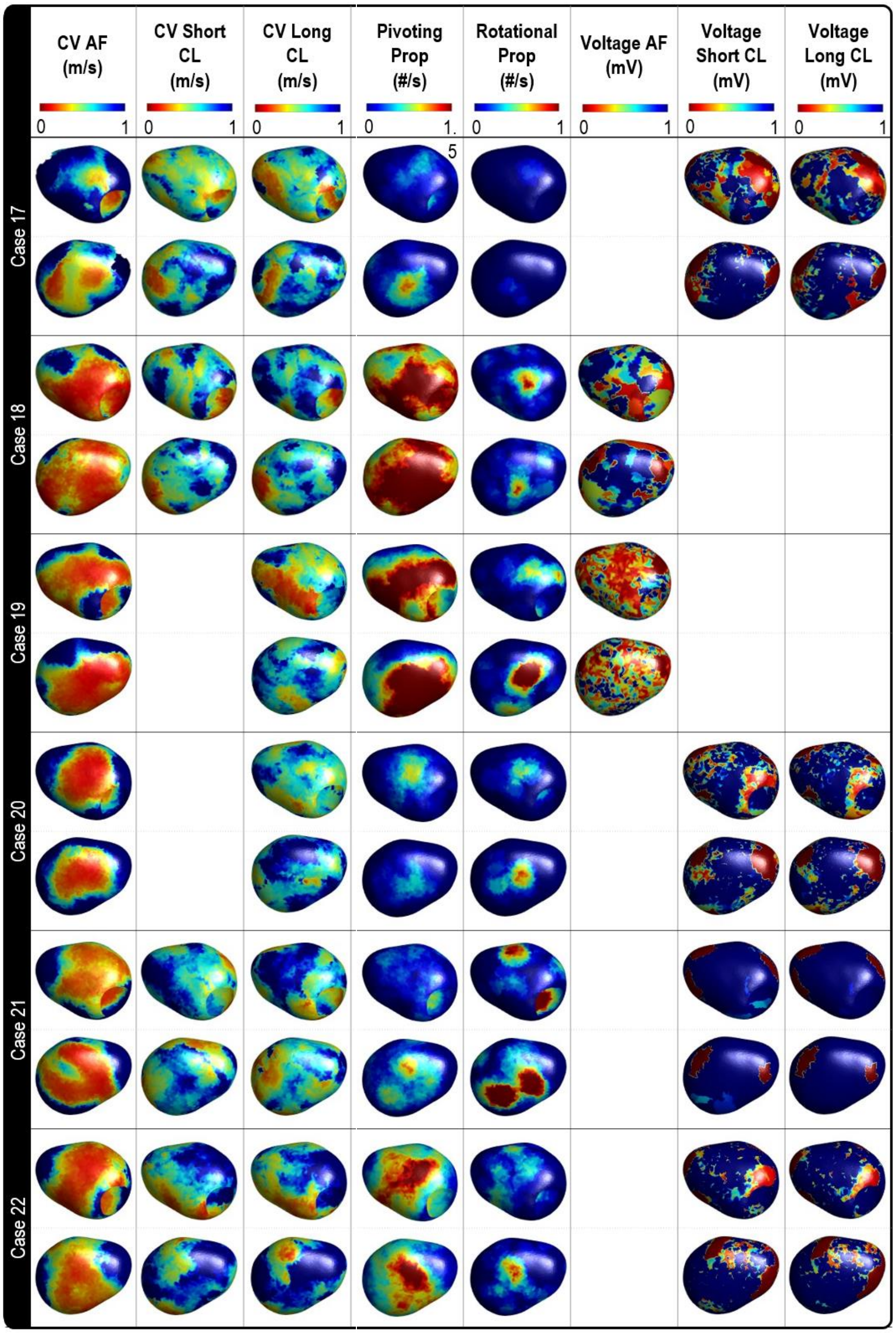

Figure S5. **Individual spatial distributions.**

Supplementary Figure legends

Figure S1. **Individual registrations with template geometry.**

Iterative Closest Point registrations of individual CDM (blue) and EAVM (red) geometries with template left atrial (grey) geometry produced from statistical shape model of all twenty-two CDM geometries. CDM: charge density mapping; EAVM: electroanatomic voltage mapping.

Figure S2. **Non-amiodarone taking population spatial distributions of CDM propagation patterns.**

Anterior (left) and posterior (right) views of CDM propagation patterns when applied to template left atrial geometry. MV: mitral valve.

Figure S3. **Non-amiodarone taking population spatial distributions of voltage.**

Anterior (left) and posterior (right) views of voltage depending on mapping protocol when applied to template left atrial geometry. MV: mitral valve.

Figure S4. **Non-amiodarone taking population spatial distributions of conduction velocity.**

Anterior (left) and posterior (right) views of conduction velocity depending on mapping protocol when applied to template left atrial geometry. MV: mitral valve.

Figure S5. **Individual spatial distributions.**

Anterior (top) and posterior (bottom) spatial distributions of measured variables for all individuals. AF: atrial fibrillation; CL: cycle length; CV: conduction velocity; Prop: propagation.
